# Corrosion Behavior and Mechanical Properties of 30CrMnSiA High-Strength Steel under an Indoor Accelerated Harsh Marine Atmospheric Environment

**DOI:** 10.3390/ma15020629

**Published:** 2022-01-14

**Authors:** Ning Li, Weifang Zhang, Hai Xu, Yikun Cai, Xiaojun Yan

**Affiliations:** 1 School of Energy and Power Engineering, Beihang University, Beijing 100191, China; guess_lining@buaa.edu.cn (N.L.); buaa_duo@buaa.edu.cn (H.X.); yanxiaojun@buaa.edu.cn (X.Y.); 2 School of Reliability and Systems Engineering, Beihang University, Beijing 100191, China; 08590@buaa.edu.cn; 3 School of Aeronautic Science and Engineering, Beihang University, Beijing 100191, China; 4 School of Aeronautics and Astronautics, Sichuan University, Chengdu 610065, China

**Keywords:** 30CrMnSiA high-strength steel, acceleration test, marine atmospheric corrosion behavior, mechanical property degradation

## Abstract

In this work, the corrosion behavior and mechanical properties of 30CrMnSiA high-strength steel under a harsh marine atmosphere environment were systematically studied using accelerated test technology, along with corrosion kinetic analysis, microstructure and phase composition analysis, electrochemical measurements, and mechanical property tests. The influence of corrosion time on corrosion kinetics was characterized by the weight loss method. The corrosion layer and its product evolution were analyzed by SEM, EDS, XRD, and XPS. The corrosion behavior of steel was studied by a potentiodynamic polarization curve and EIS. Finally, the influence of corrosion on mechanical properties was studied by tensile and fatigue tests. The results show that 30CrMnSiA high strength steel has good corrosion resistance in a harsh marine atmosphere environment. Its corrosion behavior is cyclical: the outer rust layer exfoliated, the inner rust layer became the outer rust layer, and the matrix became inner rust due to the attack by the corrosive medium. The rust layer had a great protective effect on the matrix. The mechanical properties of 30CrMnSiA high-strength steel were reduced under the corrosive environment, and corrosion had a significant effect on its fatigue resistance.

## 1. Introduction

The atmospheric corrosion of metals is very common and causes substantial economic losses every year [1]. Corrosion costs amount to at least 4–5% of the GDP, with atmospheric corrosion making the major contribution to this cost [2]. Atmospheric corrosion has been reported to account for more failures in terms of cost and tonnage than any other type of material degradation process [3]. In order to explore the atmospheric corrosion of metal materials, atmospheric corrosion exposure test stations have been established around the world, and abundant experimental data have been accumulated [4]. As one of the most used metals in an open-air exposure environment, the corrosion of steel in the atmospheric environment, especially in marine atmospheric environments, is a subject of extensive research [5,6]. With the gradual development and utilization of the ocean by human beings, the corrosion of steel in the marine atmosphere has gradually entered the field of view of researchers. However, the emphases of these studies are different, such as the long-term corrosion behavior of steel in marine atmospheric environments [7], the influence of alloying elements and their content on the marine atmospheric corrosion resistance of steel [8], and the mechanical properties and failure behavior of steel in marine atmospheric environments [9,10].

30CrMnSiA high-strength steel is widely used as a structural material in transportation, aviation, infrastructure, and other fields due to its excellent mechanical properties, such as its high strength, good toughness, good machining performance, and excellent fatigue resistance [11,12]. Atmospheric corrosion is produced by the drying and wetting cycle on the metal surface [13]. In marine atmospheric environments, the deposition of salts (NaCl and MgCl_2_) on metal surface reduces the relative humidity of the liquid film formation on it [14]. The liquid film is rich in corrosive ions such as Cl^−^, which leads to atmospheric corrosion easily in this environment. At the same time, as Cl^−^ attacks the steel matrix, it will form a unique iron hydroxide in the marine atmosphere, akaganeite (*β*-FeOOH) [15]. As a result, compared with atmospheric environments in other areas, the phase composition of a rust layer on a steel surface in a marine atmospheric environment is more complex [16], and the protective effect of rust layers on steel matrices needs to be further studied.

In order to establish an atmospheric corrosion database of steel, many corrosion test sites have been established around the world to conduct outdoor exposure tests. Morcillo et al. reviewed the extensive information on the atmospheric corrosion of weathering steel [4], and Dong et al. introduced atmospheric corrosion research in China [17]. However, the construction of the site and the development of outdoor exposure tests require considerable expense and time (ranging from months to years) [18]. Therefore, it has become a trend to develop laboratory-accelerated tests to simulate the atmospheric corrosion of steel in real environments. Guo et al. studied the effect of salts and their mixing ratio on the corrosion behavior of 316 stainless steels in a simulated salt lake atmosphere by using a wet-dry alternated test, and the results indicated that mixing different salts causes differences in the corrosion rate and corrosion products [14]. Calero et al. proposed an accelerated cyclic laboratory test based on immersion (4.2 min) in a 3.5% NaCl solution followed by drying (12 min) under infra-red lamps to evaluate the multilayered rust on carbon steel in relatively short times [19]. Sugae et al. explored the corrosion behavior of Sn-bearing steel in wet–dry environments containing chloride ions, and the results showed that the corrosion rate in the solution containing chloride ions during the drying stage was higher than in the solution containing sulfate ions [20]. Due to the wet–dry cycling characteristics on the metal surfaces, any accelerated test should take this aspect into consideration in order to ensure the consistency of the corrosion mechanism [21]. On the one hand, the corrosion resistance [22] and mechanical properties [10] of metals can be rapidly evaluated through short-term accelerated test results, as well as the protective effect of the rust layer [23], and the influence of environmental factors on both of them [24,25]. On the other hand, long-term corrosion results can be predicted from short-term test results [26,27]. However, the corrosion behavior of 30CrMnSiA high-strength steel under marine atmospheric environments, especially with high temperature, high humidity, and high chloride ion deposition rate. Furthermore, the relationship between the corrosion behavior of steel and the degradation of its mechanical properties needs to be explored.

In this paper, the corrosion behavior and mechanical properties of 30CrMNSiA high-strength steel under an indoor accelerated harsh marine atmosphere environment were systematically discussed through accelerated tests. The effect of corrosion time on corrosion kinetics was characterized by the weight loss method. The corrosion layer and its phase evolution were analyzed by scanning electron microscopy (SEM), energy dispersive spectroscopy (EDS), X-ray diffraction (XRD), and X-ray photoelectron spectroscopy (XPS). The corrosion behavior of steel was studied by a potentiodynamic polarization curve and electrochemical impedance spectroscopy (EIS). Finally, the influence of corrosion on the mechanical properties of steel was monitored by tensile and fatigue tests.

## 2. Experimental Procedures

### 2.1. Materials and Alternate Immersion Test

**Figure 1:**
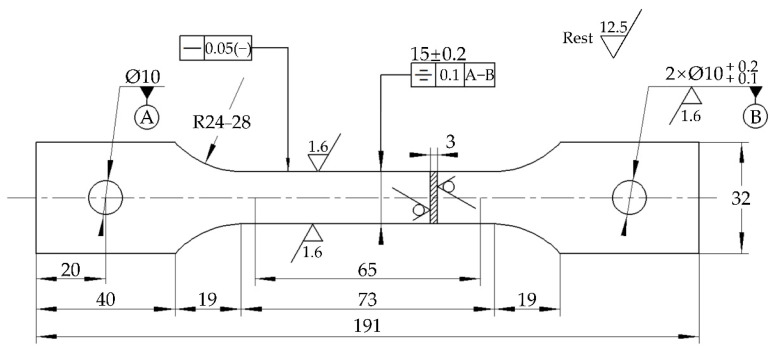
The dimensions of the tensile and fatigue specimens (unit: mm).

The chemical composition of 30CrMnSiA high-strength steel is shown in Table 1. Large specimens (50 × 50 × 3 mm; surface roughness: 1.6) were used for corrosion kinetic analysis, surface morphology observation, and rust layer composition identification, while small specimens (10 × 10 × 3 mm; surface roughness: 1.6) were used for electrochemical measurements of corrosion products. After welding a wire on one side of the small specimen, the specimen and the weld were embedded in epoxy resin. The other side of the small specimen (10 × 10 mm) was exposed and polished to a surface roughness of 1.6. Specimens for mechanical property testing were machined according to ISO 6892-1:2009 and ISO 1099:2006 standards, and the dimensions are shown in Figure 1. All the specimens were cleaned with acetone, rinsed with distilled water, and dried in a drying oven. All the specimens were photographed and prepared for the alternate immersion test.

The alternate immersion test was formulated referring to ISO 11130:2017. The ambient temperature of the test was 40 ± 1 °C, and the relative humidity was 85% ± 1%. Specimens were cyclically exposed to air for 45 min and immersed in the solution that simulated seawater for 15 min. In order to simulate a harsh marine atmosphere environment, the mass fraction of Cl^−^ in the solution based on ISO 11130:2017 was increased to 4% under the condition of maintaining various salt ratios, with the chemical composition as listed in Table 2. The pH value of the solution was modulated to 8.2 with a 0.1 mol/L NaOH solution. The test periods were 48, 96, 144, 288, 432, and 576 h. The specimens (except the small ones) were taken out and placed in a drying oven and dried at 85 °C, for 2 h before subsequent testing.

### 2.2. Corrosion Kinetic Analysis

In this work, the test specimens were cleaned first and then measured by a digital balance to record the weight (*M_i_*) before the alternate immersion test. According to the ISO 8407:2009 standard, the specimens were cleaned in an HCl solution (mixed with 80 g/L Sb_2_O_3_ and 50 g/L SnCl_2_) to remove the corrosion products after the alternate immersion test. Subsequently, the specimens were rinsed with acetone and dried in the drying oven. After that, the specimens were weighed again (*M_j_*). Each measurement was repeated three times and calibrated with blank specimens. The corrosion depth (*D*) was calculated using the following equation:

(1) $$D=\frac{M_{j}-M_{i}-M_{b}}{\rho A}$$

where *M_j_* and *M_i_* are the weights (g) of the specimen before and after the corrosion test, respectively, and *M_b_* is the mass loss of the blank specimens in the pickling process. *ρ* is the density of the steel (7.85g/cm^3^), and *A* is the exposed surface area (cm^2^).

The corrosion rate (*v*_corr_) was calculated using the following equation:

(2) $$v_{\mathrm{corr}}=\frac{D}{t}$$

where *D* is the corrosion depth (mm) of the specimen, and *t* is the corrosion time (h). The units of the corrosion rate (*v*_corr_) were eventually converted to μm/a.

### 2.3. Surface Morphology Observation and Composition Identification

After the alternate immersion test, micrographs of the surface film were performed by SEM (Zeiss Supra55) (Carl Zeiss Meditec AG, Jena, Germany) with EDS (Oxford X-ray spectrometer) (Oxford Instruments plc, Abingdon, UK). The composition of the rust powder scraped from the surface of the specimens with a standard knife was analyzed by XRD (Bruker AXS D8 ADVANCE) (BRUKER AXS GmbH, Karlsruhe, Germany) with Co *K_α_* radiation (*λ* = 0.179026 nm), which was carried out at a voltage of 40 kV and a filament current of 40 mA. The incident angle range was 10° to 90° with a scanning rate of 5°/min. The XRD data were processed by X’Pert HighScore Plus software (version 3.0) and the RIR method. Because XRD technology has limitations when distinguishing magnetite (Fe_3_O_4_) and maghemite (*γ*-Fe_2_O_3_), XPS (Thermo EscaLab 250XI) was applied to investigate the form of Fe elements in the rust powder with an Al *K_α_* (1486.6 eV) X-ray source at 14.8 kV and 1.6 A. To avoid the charge effect, the binding energy was calibrated by the C1s peak (284.8 eV), and XPS Peak 1 software was then used to perform peak fitting.

### 2.4. Electrochemical Measurement

The corrosion behavior was characterized by electrochemical impedance spectroscopy (EIS) and potentiodynamic polarization. Prior to the test, specimens were immersed in a 3.5 wt % NaCl solution for several minutes to obtain a stable open-circuit voltage. Both the electrochemical measurements were performed using a conventional three-electrode cell at room temperature on a DH7000 workstation (Jiangsu Donghua Analytical Instruments Co., Ltd., Jingjiang, China). The working electrode was the small specimens, the reference electrodes were Ag/AgCl/KCl (Sat’d), and the counter electrode was a platinum sheet electrode. EIS was measured with a perturbation amplitude of 10 mV with respect to the open-circuit potential (OCP), and the frequency range was from 10^5^ to 10^−2^ Hz. ZSimpWin software (version 3.60) was used for the data fitting of the impedance spectra. The potentiodynamic polarization curves of the specimens were recorded in the applied potential range from −0.5 to 0.5 V_vs OCP_ at a scan rate of 1 mV/s.

### 2.5. Mechanical Property Test

In order to characterize the mechanical property degradation of the 30CrMnSiA high-strength steel in the simulated harsh marine atmosphere environment, tensile tests and fatigue tests were carried out by a hydraulic MTS testing machine (MTS Systems Corporation, Eden Prairie, MN, USA). The tensile test was carried out at room temperature with a loading speed of 1 mm/min. The tensile strength, yield strength, and elongation at break were selected to evaluate the mechanical properties. For the fatigue tests, the specimens were tested under constant amplitude cyclic loading at a maximum stress (*σ*_max_) of 768.9 MPa until fractured. The applied stress ratio (*R*=*σ*_max_/*σ*_min_) was 0.1. The loading wave form was sinusoidal, and the loading frequency was 10 Hz. The fatigue fractography was observed by SEM, and the effects of simulated marine atmospheric corrosion on the fatigue life of 30CrMnSiA high-strength steel were analyzed.

## 3. Results and Discussion

### 3.1. Corrosion Kinetic Analysis

Figure 1a,b show the corrosion depth and corrosion rate (*v*_corr_), respectively, which were obtained from the corrosion kinetic analysis of the specimens after the alternate immersion test for 576 h.

**Figure 2:**
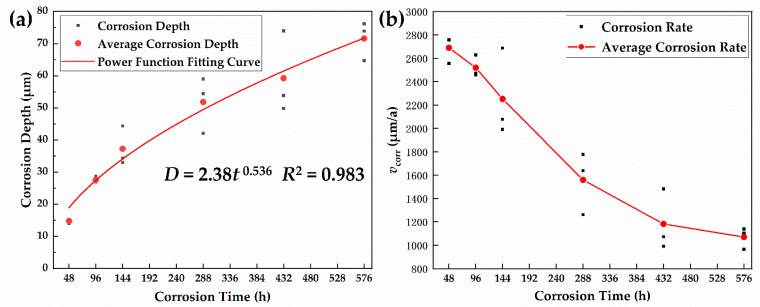
Corrosion Kinect analysis after a 576-h alternate immersion test: (**a**) calculated corrosion depth; (**b**) corrosion rate.

As shown in Figure 2a, the corrosion depth of the corrosion products was increased, whereas the increment was reduced with prolonged corrosion time. It is widely accepted that the atmospheric corrosion of steel conforms to the power function model of the following type: *D* = *At^n^* [17]. The corrosion depth data in Figure 2a has been fitted with a power function model, and the fitting curve is shown as the red line. The regression model of the corrosion depth data of the 30CrMnSiA steel with corrosion time is as follows:

(3) $$D=At^{n}=2.38t^{0.536}$$

where *D* represents the corrosion depth of steel (μm), t is the corrosion time (h), and *A* and n are constants. The parameter *A* can be considered as a measure of the initial corrosion resistance of the steel, while *n* reflects the development trend of corrosion [28]. Generally, when *n* < 1, corrosion is decelerating, and the rust layer of the material is protective to the matrix [29]. In a previous study, the *n* values of the two steels (Q235 and Q450NQR1) exposed in a tropical marine zone of the Spratly Islands were both greater than 1, which means that both corrosion processes exhibit accelerated trends [30]. Meanwhile, the value of *n* is inversely proportional to corrosion resistance [8]. The corrosion resistance here can also be understood as the protective effect of the rust layer on the matrix. Wu et al. studied the synergy of Cu and Sb to enhance the resistance of 3%Ni weathering steel under a marine atmospheric environment. The results showed that the *n* value of the BM steel was close to 1 (0.964), suggesting an almost powerless resistance to corrosion, but after adding 0.5%Cu and 0.2%Sb in the steel, *n* changed to 0.687, which implies a significant improvement in the corrosion resistance after Cu and Sb addition [8]. In contrast, from the fitted data, *n* is 0.536, which means that the rust layer of 30CrMnSiA high-strength steel under a marine atmospheric environment has an obvious protective effect on the matrix.

The average corrosion rate decreased rapidly during the first 288 h and then gradually slowed down. From 2014 to 2018, our research group carried out 30CrMnSiA high-strength steel exposure tests at the Wanning Outdoor Exposure Test Station. Wanning is a typical marine atmospheric corrosive environment, and the environmental information is shown in Table 3. The results show that the average corrosion rates of steel in the natural marine atmosphere were 379.3548, 363.1173, and 314.809 μm/a, respectively. The average corrosion rate was more than 1000 μm/a during the whole process of the alternate immersion test, which proves that the test has a good acceleration effect and can simulate harsh marine atmosphere environments.

### 3.2. Surface Microstructure Analysis

#### 3.2.1. Surface Morphologies of Corrosion Products

**Figure 3:**
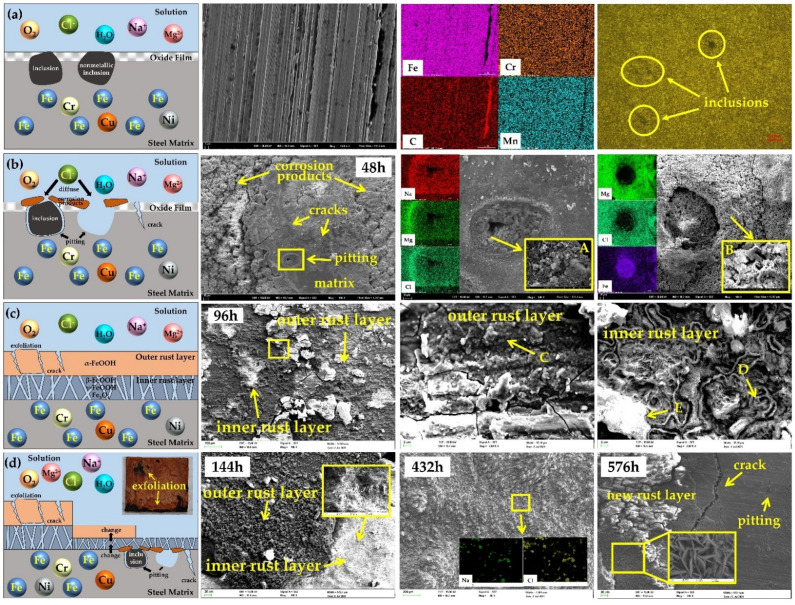
Corrosion behavior diagram with SEM and EDS analysis: (**a**) blank; (**b**) pitting; (**c**) exfoliation; (**d**) corrosion cycle.

Combining the observation of surface microscopic morphology and EDS results, the corrosion behavior of the 30CrMnSiA high-strength steel under the indoor accelerated harsh marine atmosphere environment is shown in Figure 3.

Figure 3a shows that the surface of the uncorroded specimen was covered with parallel machining traces. It is easy to accumulate Cl^−^ in the grooves of machining traces, which may lead to the occurrence of corrosion. The distribution of elements analysis on the surface by EDS revealed a large number of elongated and spot-like non-metallic inclusions. A metallographic examination of the blank specimen revealed substantial inclusions on the surface of the steel, where corrosion began [31]. It has been widely mentioned in previous studies that chloride ions can significantly affect the development of corrosion [32,33]. In marine atmospheric environments, thin liquid films rich in corrosive anions such as Cl^−^ will form on the surface of steel, and Cl^−^ will preferentially adsorb and accumulate at high energy positions such as inclusions. Cl^−^ not only has electrical conductivity, but also can induce the pitting of passivated metals, promoting the development of pitting [34]. Generally, there are two kinds of inclusions in steel: anode inclusions and cathode inclusions. The corrosion mechanisms of steel that they cause are different [35,36]. The potential of anodic inclusions is more negative than the potential of the oxide film on the surface of steel. When a liquid film is produced on the surface, the inclusions will dissolve completely or partially, forming micro-cavities on the surface of steel. Therefore, cavitation can be seen in the pitting corrosion of the anode inclusion in SEM observation. Cl^−^ will then accumulate in the micro-cavity and form a closed acidified autocatalytic battery, which leads to the continuous growth of the micro-cavity and finally leads to pitting. Cathode inclusions are more stable in a corrosive environment due to their positive potential and appear as cathodes in the pitting process. At this time, pitting occurs between the inclusion and the steel matrix. In SEM observations, a gap can be seen in the steel matrix around the inclusion and gradually develops into pitting, accompanied by a large number of corrosion products attached to the pitting.

As shown in Figure 3b, a large number of cracks appeared on the surface of the specimen after corrosion for 48 h, and corrosion products began to accumulate at the same time. The source of the cracks may be the stress caused by the corrosion products and the surface strength decline caused by the dissolution of grain boundaries on the steel surface. Two kinds of pitting can be observed on uncorroded metal matrices. Around the anodic pitting, there were some cigar-shaped crystals typical of akaganeite with fine plates of lepidocrocite (*γ*-FeOOH) with a “flowery” structure, marked as A [37]. EDS analysis shows that MgCl_2_ and NaCl were enriched around the pitting. Liu et al. [38] studied the influence of different hard metals on the corrosion of carbon steel in a chloride ion atmospheric environment and found that Mg^2+^ was greater than Na^+^ and K^+^ in terms of corrosion rate and corrosion current density, proving that Mg^2+^ had a catalytic effect on the corrosion of steel. On the other hand, in an environment containing Mg^2+^, the relative content of akaganeite was higher, and the protection ability of the rust layer was inferior to an environment containing Na^+^ and K^+^. Many corrosion products were found around the negative pitting, and a massive amount of chloride was deposited on the cotton ball akaganeite (*β*-FeOOH), marked as B [37], in the microscopic morphology. In marine atmospheres, due to the enrichment of Cl^−^, a special corrosion product, akaganeite, is produced on steel, which not only accelerates the corrosion rate but also reduces the protection ability of the rust layer [39,40,41]. EDS analysis showed that there are quantities of MgCl_2_ deposited on the surface corrosion products, which is due to the low solubility and precipitation at the low critical relative humidity of MgCl_2_. A large number of pits and cracks on the surface of the specimen provided channels for corrosive media (H_2_O, O_2_, and Cl^−^) to pass through the oxide layer of the steel and attack the steel matrix, and further accelerated the steel from pitting to the uniform corrosion stage.

After corrosion for 96 h, the rust layer on the specimen surface showed obvious stratification, as shown in Figure 3c. A large number of globular characteristics typical of goethite (*α*-FeOOH), marked as C [37], existed in the outer rust layer. Plenty of cracks and even exfoliation occurred on the outer rust layer, where the exposed inner rust layer contained doughnut-shaped formations typical of magnetite (marked as D), spongy whitish formations typical of akaganeite (marked as E) [42], and fine plates of lepidocrocite. Goethite in the outer rust layer protected the steel matrix to some extent, and this protection disappears after exfoliation. Akaganeite in the inner rust layer has the strongest oxidation activity among these iron oxides, so it is easily reduced in the wetting stage to form amorphous intermediate products, thus accelerating the corrosion of steel [42]. The inner rust layer is exposed in the atmosphere due to the exfoliation of the outer rust layer, and its corrosion products provide a channel for the corrosive medium to pass through the rust layer and reach the steel matrix due to the characteristics of the structure.

Thus, the atmospheric corrosion of steel enters a cycle: Firstly, the surface of the matrix gradually becomes the inner rust layer due to the occurrence of corrosion. The inner rust layer then grows and becomes the outer rust layer. Finally, the outer rust layer falls off, exposing the inner rust layer to the corrosive environment and providing a channel for the corrosive medium to attack the matrix. As shown in Figure 3d, the macroscopic features of the surface of the specimens that corroded for 144, 288, 432, and 576 h were basically the same, and the outer rust layer showed exfoliation behavior. There were substantial chloride particles deposited on the surface of the rust layer, as shown by the microscopic morphology of the outer rust layer on the specimen that corroded for 432 h, which proves the protective effect of the outer rust layer on steel. At the same time, it can be seen that the outer rust layer of the specimen that corroded for 432 h is denser than that of the specimen that corroded for 96 h, but the area of exfoliation is also larger. In the microscopic morphology observation of the specimen after 144 h of corrosion, a large number of fine plates of lepidocrocite can be observed in the inner rust layer, indicating that the matrix in this region has been corroded for a period of time. With the continuous development of corrosion, this part of the inner rust layer will gradually become the outer rust layer. In addition, the inner rust layer with fine plates of lepidocrocite was observed in the microscopic morphology of the specimen that corroded 576 h after the exfoliation of the outer rust layer. After removing part of the inner rust layer, the matrix of the specimen could be observed, and there were some pits and cracks on the surface of the matrix. By studying the corrosion behavior of 30CrMnSiA high-strength steel under an indoor accelerated harsh marine atmospheric environment, it was demonstrated that, although the corrosion is uniform, corrosion occurs at different stages in the fine area of the exposed surface, which leads to specimen thinning due to the exfoliation of the outer rust layer. The surface of the matrix was also rugged due to pits and cracks. This had a fatal effect on the mechanical properties of the specimens, as will be explained in the following sections.

#### 3.2.2. Composition of the Rust Layer

**Figure 4:**
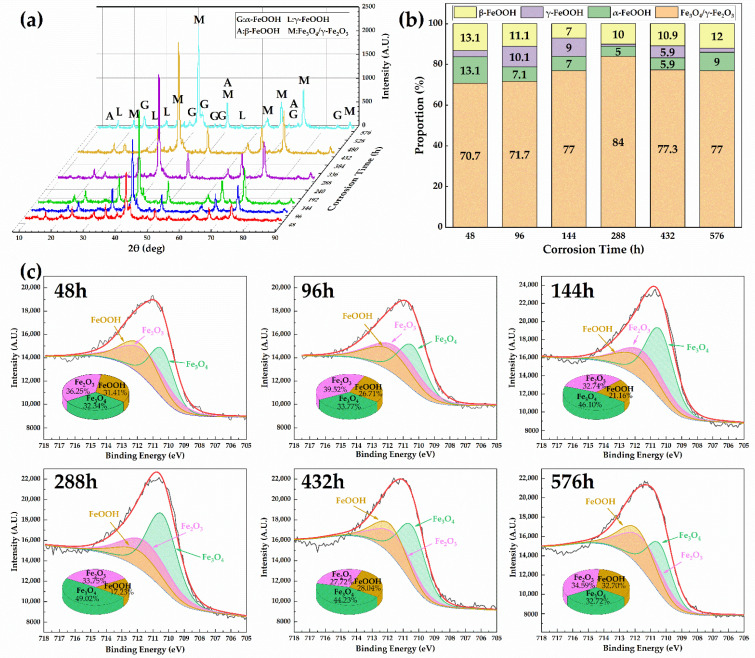
X-ray diffraction analysis of rust formed on surfaces of 30CrMnSiA steel: (**a**) phase compositions; (**b**) the relative proportion of each phase; (**c**) XPS spectra of Fe2p3/2 and the atomic percentage of each phase in the rust film for 48, 96, 144, 288, 432, and 576 h.

Under different corrosion times, XRD and RIR methods [43] were used to analyze the rust components and the relative content of each phase on the surface of the 30CrMnSiA high-strength steel, as shown in Figure 4. Previous studies have shown that the rust of steel in a marine atmosphere always contains three kinds of iron hydroxides—goethite (*α*-FeOOH), akaganeite (*β*-FeOOH), and lepidocrocite (*γ*-FeOOH)—as well as the iron oxides magnetite (Fe_3_O_4_) and magnetite (*γ*-Fe_2_O_3_), which have the strongest diffraction peak [37,44,45].

Figure 4a shows that the main composition of the rust layer of the 30CrMnSiA high-strength steel under an indoor accelerated harsh marine atmospheric environment is the same as that in an outdoor exposure test in a marine atmospheric environment, which is mainly Fe_3_O_4_/*γ*-Fe_2_O_3_, *α*-FeOOH, *β*-FeOOH, and *γ*-FeOOH. Analyzing the strongest diffraction peak, the content of Fe_3_O_4_/*γ*-Fe_2_O_3_ increased gradually with the occurrence of corrosion and stabilized after corrosion for 144 h.

Figure 4b shows the relative content of each phase in rust at different corrosion times. Since Fe_3_O_4_ and *γ*-Fe_2_O_3_ are difficult to distinguish by XRD, the total mass fraction of Fe_3_O_4_/*γ*-Fe_2_O_3_ is given [46]. The corrosion products contain more than 70% of relatively dense Fe_3_O_4_ with a certain anionic selection ability and 5–13% of *α*-FeOOH with a dense structure, which can slow down corrosion to a certain extent. This is the same as the result of microscopic morphology observation. Since the relative content of each product changes dynamically with the corrosion time, some scholars use the protective index *PAI* to evaluate the protective effect of the rust layer on the steel matrix [47]. The protective index *PAI* [48] is defined as the ratio of α* (the sum of the relative content of *α*-FeOOH and Fe_3_O_4_/*γ*-Fe_2_O_3_) to γ* (the sum of the relative content of *γ*-FeOOH and *β*-FeOOH), as shown in Equation 4. When this protective index of the rust layer is close to or over 2, the rust layer has good protective effects [30]. Figure 4b shows that the protective index *PAI* values of the 30CrMnSiA high-strength steel’s rust layer were all above 4, indicating that the rust layer has a good protective effect on the steel matrix. Simultaneously, the *β*-FeOOH content was not low in the corrosion products at all stages of the test, which proves that Cl^−^ in the marine atmosphere plays an important role in the corrosion of steel.

(4) $$\mathrm{PAI}=\frac{\alpha^{*}}{\gamma^{*}}=\frac{\alpha -\mathrm{FeOOH}+{\mathrm{Fe}}_{3}O_{4}/\gamma -{\mathrm{Fe}}_{2}O_{3}}{\gamma -\mathrm{FeOOH}+\beta -\mathrm{FeOOH}}$$

To further distinguish the content difference between Fe_3_O_4_ and *γ*-Fe_2_O_3_, XPS technology was used to study the rust components. The XPS spectra of Fe2p3/2 in the rust layer are shown in Figure 4c. It can be seen that the spectrum of Fe2p3/2 contains three peaks at 710.4eV [49], 711.4eV [50], and 711.8eV [51], corresponding to Fe_3_O_4_, *γ*-Fe_2_O_3_, and FeOOH, respectively. The relative content of each phase was calculated by the area of the peak splitting curve, and the results are also shown in Figure 4c. With the increase in corrosion time, the content of Fe_3_O_4_ gradually increased, the content of *γ*-Fe_2_O_3_ basically remained unchanged, and the content of FeOOH gradually decreased. On one hand, the high content of Fe_3_O_4_ made the corrosion products denser and prevented anions from attacking the steel matrix; on the other hand, the inhibition of the corrosion products on corrosion was gradually enhanced. This is all consistent with the previous results.

### 3.3. Electrochemical Tests

Because atmospheric corrosion is a process of failure caused by electrochemical reactions between metal and its surrounding atmospheric environment, electrochemical tests are effective methods to evaluate the corrosion mechanism of steel [52]. In order to investigate the corrosion behavior of 30CrMnSiA high-strength steel in a simulated harsh marine atmospheric environment for different periods, electrochemical impedance spectroscopy (EIS) and potentiodynamic polarization curves were performed.

#### 3.3.1. Potentiodynamic Polarization Curves

**Figure 5:**
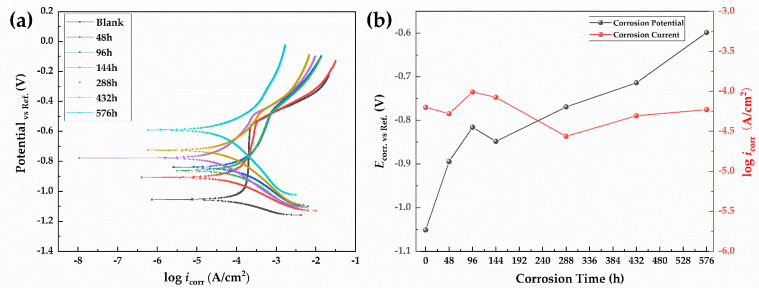
Potentiodynamic polarization curves (**a**) and corrosion potential (**b**) of 30CrMnSiA steel.

The potentiodynamic polarization curves of the 30CrMnSiA steel at different times are shown in Figure 5a. The corrosion potential (*E*_corr_) and the corrosion current density (*i*_corr_) was obtained from extrapolation of the Tafel curves, as shown in Figure 5b. Other parameters derived from Tafel curves are shown in Table 4.

With the increase in corrosion time, the corrosion potential gradually shifted to the positive direction, from −1.05 to −0.5983 V. This means that the corrosion products on the surface of the specimens inhibited the corrosion. In the early stage of the test, an obvious passivation zone and pitting potential were found in the anodic zone of the polarization curve. With the increase in corrosion time, the passivation area gradually disappeared. The corrosion changed from pitting to uniform corrosion. It is obvious that the corrosion current first rose and then declined. The corrosion current began to rise from 0 h and then reached the maximum value of 98.4011 μA/cm^2^ at 96 h. The corrosion current then began to decrease, reached a minimum value of 27.4536 μA/cm^2^ at 288 h, and then gradually stabilized. The similar shapes of the polarization curves show that the corrosion mechanism of the steel did not change. The cathodic process was controlled by the charge transfer during the rust layer reduction reaction, and the anodic process was controlled by the iron dissolution reaction [40]. In the equipotential case, in the cathodic process, with the development of corrosion, its current presented an increasing trend, indicating that the rust layer involved in the reduction reaction was gradually increasing. In contrast, in the anodic process, its current showed a decreasing trend, indicating that the iron dissolution reaction was gradually reduced, which further explains the protection mechanism of the rust layer. A similar conclusion can be obtained from the gradually increasing *β*_a_. Therefore, throughout the whole test process, the current in the cathode process gradually increased, while the current in the anode process gradually decreased, resulting in the fluctuation of the corrosion current of the corrosion system within 100 μA. This indicates that the corrosion mechanism of the 30CrMnSiA high-strength steel did not change significantly in the test, and the positive shifting of the corrosion potential reflected the accumulation of the corrosion products and their influence on the whole corrosion system. The polarization curve analysis results also confirm the above results.

#### 3.3.2. Electrochemical Impedance Spectroscopy (EIS)

**Figure 6:**
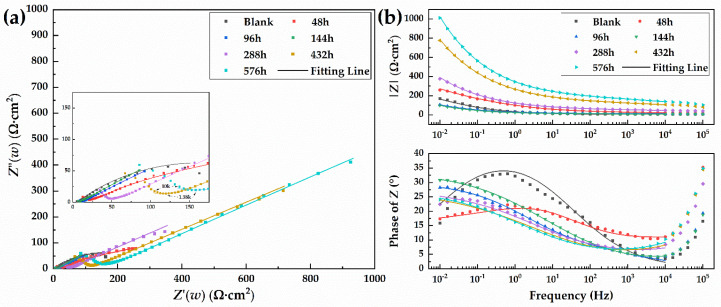
Nyquist plot (**a**) and Bode plot (**b**) of 30CrMnSiA steel for different corrosion periods.

The EIS measurement results of the 30CrMnSiA steel under different corrosion times are shown in Figure 6. As the Nyquist plots show in Figure 6a, the electrochemical impedance curve of the blank specimen consists of a small capacitive loop at the intermediate frequency region, and a Warburg impedance at the low frequency region appeared on the electrochemical impedance curve at 48 h. Subsequently, as the corrosion products build up, the curve should contain two capacitance loops from the double capacitance layer and the rust layer at the intermediate frequency region, as well as a Warburg impedance in the low frequency region. The appearance of Warburg impedance indicates that the electrode process changes from the charge transfer process to the mass transfer process of corrosion products. In the Nyquist diagram, the high frequency region is related to the charge transfer process of the double electric layer, reflecting the inhibition effect of the rust layer on corrosion. The low frequency region is related to the diffusion process of electrolytes through the rust layer, reflecting the electrode process between the rust layer and the metal interface [53].

The characteristic frequencies of capacitive loops and Warburg impedance are presented according to the Bode plots, as shown in Figure 6b. In the Bode diagram in Figure 6b, the |*Z*| of the low frequency region fluctuates around 200 Ω·cm^2^ in the first 144 h of corrosion, then increases with the increase in corrosion time and reaches a maximum value of 1000 Ω·cm^2^ after 576 h of corrosion. This phenomenon shows that, in the early stage of corrosion, the rust layer has little resistance to the penetration of the corrosive medium into the matrix. Simultaneously, the reduction in the value of |*Z*| could be ascribed to the formation of the rust layer, which may accelerate the electrochemical corrosion process on the matrix. However, with the development of corrosion, the rust layer becomes increasingly dense, and this resistance increases. In the Bode diagram, several peaks of the phase angle often represent several time constants caused by state variables in the impedance spectrum. The peak of the uncorroded specimen near 1 Hz gradually weakened and disappeared with the occurrence of corrosion, confirming the occurrence of pitting corrosion on the specimen surface and the disappearance of the passivation film. At the same time, a new peak was generated in the low frequency region, which represents the appearance of the rust layer protecting the matrix. This phenomenon illustrates the presence of the rust layer and the need to use different equivalent circuits to fit the EIS data.

**Figure 7:**
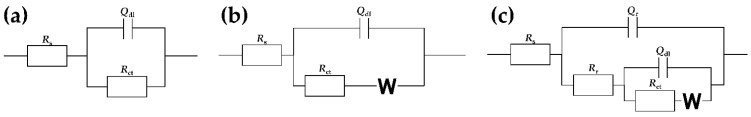
The equivalent electrical circuit for the EIS data: (**a**) blank; (**b**) 48 and 96 h; (**c**) 144, 288, 432, and 576 h.

In order to determine the impedance parameters under different corrosion times, combined with the above analysis and relevant literature [34,38], the equivalent circuit as shown in Figure 7 was used to fit the data measured by EIS. The arc at very high frequency (10^4^–10^5^ Hz) is due to the reference electrode, so the data points in the 10^4^–10^5^ Hz range were deleted in the fitting process. The components are interpreted as follows: *R*_s_ is the solution resistance without compensation, *R*_r_ and *Q*_r_ are the pore solution resistance and capacitance of the rust film, *Q*_dl_ is the double-layer capacitance, *R*_ct_ is the charge transfer resistance, and W is the diffusion-induced Warburg element. *Q* is the constant phase element (CPE) used to represent the capacitance of the double-layer and rust layers. The diffusion-induced Warburg element is known as the finite-space diffusion Warburg impedance. It represents the transition from Warburg behavior (45° line) to a capacitive behavior in the low frequency range. All circuit parameters are used to describe the interface mechanism. The error (*χ*^2^) of all the fitting results is between 10^−3^ and 10^−5^, indicating that the equivalent circuit can fit the measurement data well. The results of the fitted data are shown in Table 5.

Table 5 shows that the parameter *R*_ct_, which is used to evaluate the difficulty of charge transfer, first decreased from 313.3 to 27.18 Ω·cm^2^ and then increased to 427.7 Ω·cm^2^ at 576 h. The value of *R*_ct_ on the blank specimen indicates that there is an oxide film on the surface of the uncorroded specimen. The phenomenon that *R*_ct_ decreases first indicates that the rupture of the oxide film and the protection of the rust layer on the matrix is weak in the early stage of corrosion development, which should be attributed to the promotion of the electrochemical corrosion process on the matrix by the corrosion products. *R*_ct_ then increases significantly with the increase in corrosion time, indicating that the charge transfer of the corrosion system becomes increasingly difficult with the development of corrosion, and corrosion was inhibited by the rust layer. At the same time, the *R*_r_ value also increased significantly from 4.754 to 153.5 Ω·cm^2^, indicating that it is more difficult for the corrosion medium to reach the metal matrix, and indicating the protection of the rust layer.

### 3.4. Mechanical Property Test

#### 3.4.1. Tensile Test

**Figure 8:**
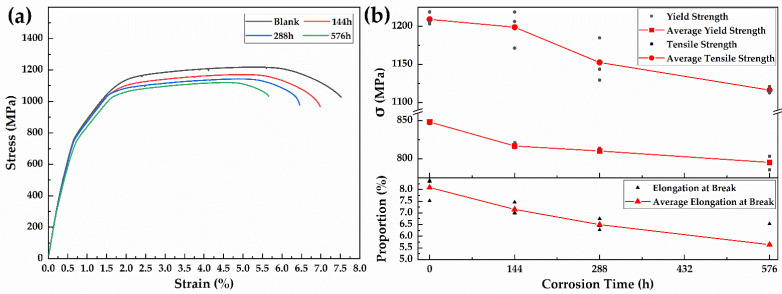
Stress–strain curve (**a**) and mechanical property parameters (**b**) of 30CrMnSiA steel after the 576-h alternate immersion test.

The tensile property degradation results of the 30CrMnSiA high-strength steel specimens after the 576 h alternate immersion test are shown in Figure 8. Figure 8a clearly shows that corrosion degrades the tensile properties of 30CrMnSiA high-strength steel. With the passage of corrosion, the tensile properties of the specimen deteriorated more seriously. Because 30CrMnSiA high-strength steel has no obvious yield plateau in the tensile test, the yield strength of the 30CrMnSiA high-strength steel is characterized by *σ*_0.2_. The data in Figure 8b show that, under corrosion times of 144, 288, and 576 h, the yield strength of 30CrMnSiA high-strength steel decreased by 3.72%, 4.49%, and 6.25%, the tensile strength decreased by 0.86%, 4.68%, and 7.69%, and the elongation at break decreased by 11.57%, 19.68%, and 30.27%, respectively. Combined with the observations of the surface morphology analyses of the specimens, it can be seen that with the progress of the test, the rust layer on the surface accumulated and when it reached a certain thickness, exfoliation occurred. Exfoliation results in a significant decrease in the cross-section area of the working section of the specimens, and the original flat surface of the specimen becomes rugged, which results in a conspicuous decrease in the tensile properties of specimens after corrosion.

#### 3.4.2. Fatigue Test

**Figure 9:**
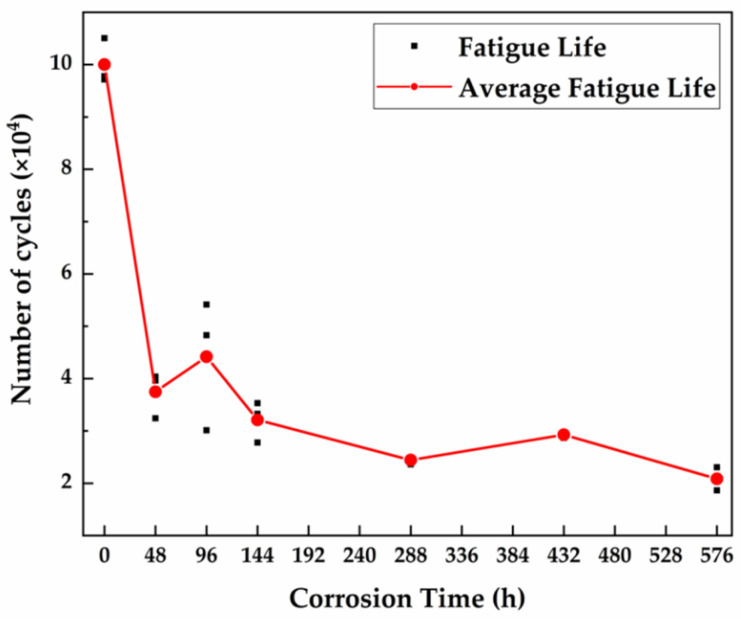
Fatigue life of 30CrMnSiA steel after the 576-h alternate immersion test.

Figure 9 shows the fatigue life of specimens under different corrosion times. The results show that the fatigue life of specimens decreases rapidly after corrosion—by more than 50%. Studies show that corrosive environments will significantly reduce the fatigue resistance of steel and ultimately fail to reach the fatigue limit [54,55]. As is known, the process of fatigue fracture includes three stages, fatigue crack initiation, fatigue crack propagation, and final rupture, and the corrosion of steel has varying degrees of influence on these three stages.

**Figure 10:**
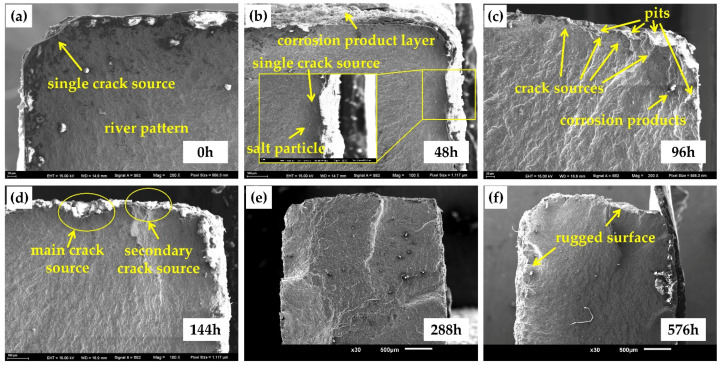
The microscopic morphology of the fatigue crack initiation region: (**a**) blank; (**b**) 48 h; (**c**) 96 h; (**d**) 144 h; (**e**) 288 h; (**f**) 576 h.

Figure 10 shows the microscopic morphology of the fatigue crack initiation region after different corrosion times. Figure 10a shows that the fatigue crack originated from the corner of the uncorroded specimen, and the crack source was single. Meanwhile, obvious river patterns were observed. After 48 h, a thin layer of corrosion products was observed on the surface of the specimen, and there were corrosion pits of different sizes and shapes. At this time, the fatigue cracks originated from a pit on the secondary surface of the specimen (the surface was the corroded product). The fatigue source area appeared semicircular, and some fine salt particles were observed. However, the fatigue crack source was still single at this time, which originated from the largest pit near the corner of the specimen, as shown in Figure 10b. After 96 h, this situation changed. Many corrosion pits helped crack initiation, and the single crack source became multiple crack sources. Converging diverging river patterns were observed, and corrosion products were also attached to the fatigue source region, as shown in Figure 10c. After 144 h, the specimen showed an exfoliation phenomenon, and the surface became rugged. In the fatigue source area, the intersection of a primary crack source from the deepest corrosion pit on the surface and a large number of secondary crack sources was observed, as shown in Figure 10d. With the further development of corrosion, because the specimen surface is more rugged, cracks no longer initiate from the corner of the specimen and may initiate from multiple corrosion points on the matrix surface, as shown in Figure 10e,f. This phenomenon makes it more difficult to judge the fatigue crack initiation region. As the initiation stage of fatigue cracks occupies the vast majority of the fatigue life of materials, and pitting has a substantial impact on the initiation of cracks [56,57], the occurrence of corrosion has a substantial impact on the fatigue life of materials, which is reflected in the test by the fact that the fatigue life of specimens was reduced by 50% as long as they were corroded.

**Figure 11:**
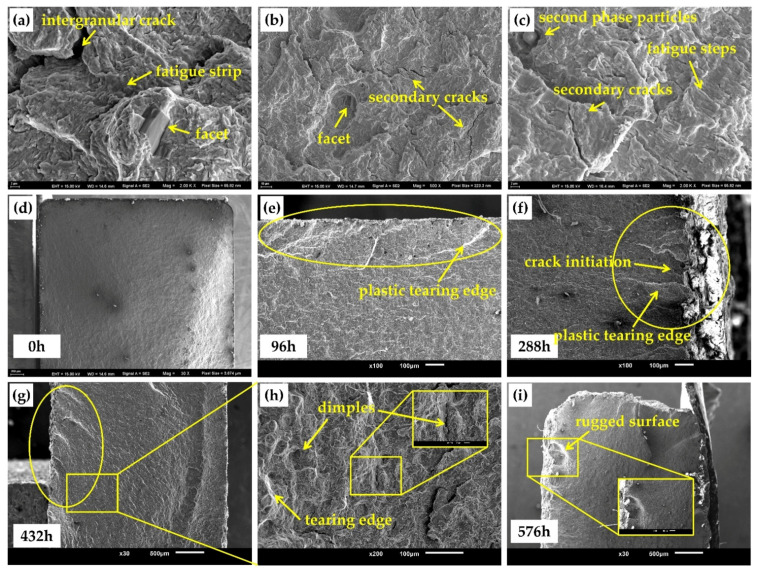
The microscopic morphology of the fatigue crack propagation region: (**a**–**c**) micromorphological characteristics; (**d**) blank; (**e**) 96 h; (**f**) 288 h; (**g**) 432 h; (**h**) yellow box of (**g**); (**i**) 576 h.

Figure 11 shows the microscopic morphology of the fatigue crack propagation region after different corrosion times. In the crack propagation region, the corrosion could not invade into the steel, so it had little influence on the micromorphological characteristics. As shown in Figure 11a–c, a large number of secondary cracks, intergranular cracks, fatigue steps, fatigue strips, and small facets can be observed in this area, which are the common microscopic morphology characteristics of the crack propagation region of high-strength steel. However, corrosion still has a negative effect on the crack propagation stage, which comes from the interlace between the specimen surface and the crack growth region. Figure 11d shows that the surface of the uncorroded specimen was flat, and cracks started from the corner and gradually expanded. However, after corrosion, the surface of the specimen became rugged, and the corrosion pits were all over the crack propagation path. When the crack expanded to the vicinity of the corrosion pits, due to stress concentration or strength shortage, the direction of the crack growth shifted, which was shown by the characteristics of irregular steps and white plastic tearing edge in the SEM observations, as shown in Figure 11e. At the same time, under the action of fatigue load, some corrosion pits also generated small fatigue cracks, which coincided with the expansion path of the main crack, as shown in Figure 11f. As the corrosion deepened, the characteristics of the steps that can be observed become more obvious, and the height of the steps was also higher, as shown in Figure 11g. By further magnifying the step characteristics, it could be found that there were many dimples and tearing edge features in the vicinity, which can often be found in the instantaneous fracture regions of fatigue, as shown in Figure 11h. These phenomena indicated that corrosion can change the propagation speed and direction of fatigue cracks and then reduce the fatigue life of specimens.

**Figure 12:**
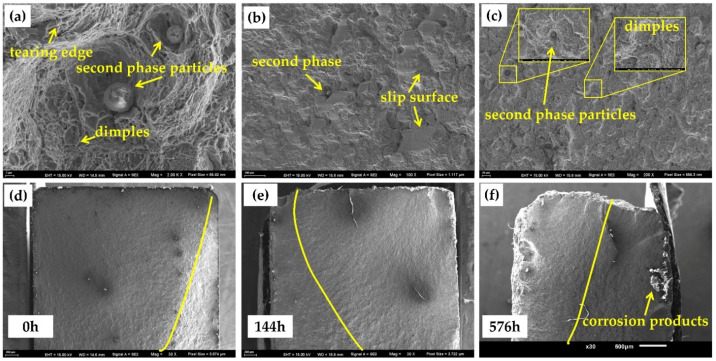
The microscopic morphology of the final rupture region: (**a**–**c**) micromorphological characteristics; (**d**) blank; (**e**) 144 h; (**f**) 576 h.

Figure 12 shows the microscopic morphology of the final rupture region after different corrosion times. For this region, a large number of tearing edges, dimples, slip surfaces, and second phase particles could be observed, as shown in Figure 12a–c. Because the corrosive medium cannot attack the internal structure of steel, the microscopic characteristics of the final rupture region were basically the same. However, due to the occurrence of corrosion, the surface of the specimen became rugged, and the cross-sectional area of the specimen decreased. Therefore, the contribution of corrosion to the final rupture region can be summed up in two aspects: On the one hand, due to the reduction in the specimen’s sectional area, the ability to cope with the same load decreased, which manifested as the increase in the area of the final rupture region in the SEM observations, as shown in Figure 12d–f. The yellow line in the figure represents the boundary between the fatigue crack propagation region and the final rupture region by artificial observation. On the other hand, the rugged surface of the specimen led to stress concentration, which further reduced the static strength of the specimen. As shown in Figure 12f, serious corrosion was observed in the final rupture region, and a large number of corrosion products invaded the surface of the specimen. The results of this section are similar to the results of the stretch test.

To sum up, corrosion will act on the three stages of fatigue fracture and then lead to a serious decline in the fatigue life of steel. As shown in Figure 10f, Figure 11i and Figure 12f, corrosion has a serious impact on all three stages of fatigue fracture. The results show that the fatigue life of specimens that has corroded for 576 h decreases by about 80%.

## 4. Conclusions

The corrosion behavior and mechanical properties of 30CrMnSiA high-strength steel subjected to an alternate immersion test for 576 h under a simulated harsh marine atmosphere environment were analyzed. Detailed study revealed the following conclusions:

1. The corrosion kinetics of the 30CrMnSiA high-strength steel followed the power function $D=At^{n}=2.38t^{0.536}$ after 576 h of corrosion, which proves that the material has good corrosion resistance. The test scheme also showed good acceleration.
2. The rust layer contains abundant goethite, magnetite, and maghemite, which have dense structures and can effectively protect the matrix from corrosive solutions.
3. The atmospheric corrosion of 30CrMnSiA high-strength steel starts from a pitting corrosion and gradually leads to exfoliation. The corrosion enters a cycle. Firstly, the surface of the matrix gradually becomes the inner rust layer due to the occurrence of corrosion, and the inner rust layer then grows and becomes the outer rust layer. Finally, the outer rust layer falls off, exposing the inner rust layer to the corrosive environment and providing a channel for the corrosive medium to attack the matrix.
4. Electrochemical tests further prove that the protective effect of the rust layer on the matrix becomes stronger with the development of corrosion and tends to be stable.
5. The tensile and fatigue properties of 30CrMnSiA decreases after corrosion. After 576 h of corrosion, the tensile strength, yield strength, and elongation at break decreased by 6.25%, 7.69%, and 30.27%, respectively. The influence of corrosion on fatigue performance was evaluated from three stages of fatigue fracture. In the crack initiation stage, which occupies most of the fatigue life of specimens, due to the pitting that occurred, the fatigue life reduced by 50%.

## Figures and Tables

**Table 1:** Chemical compositions of the 30CrMnSiA high-strength steel.

Element	C	Si	P	Mn	S	Cu	Cr	Ni	Fe
Composition (wt %)	0.29	0.98	≤0.025	0.95	≤0.025	≤0.025	1.02	≤0.30	Bal.

**Table 2:** Chemical compositions of the simulated seawater, g/L.

NaCl	Na_2_SO_4_	CaCl_2_	MgCl_2_·6H2O	KCl
37.2	6.14	1.74	16.67	1.04

**Table 3:** Environmental factor data of the Wanning Outdoor Exposure Test Station.

**Annual Mean** **Temperature (°C)**	**Time of Wetness** **(h/a)**	**Radiation Time** **(h/a)**	**SO_2_** **(mg/100 cm^2^·d)**	**NH_3_** **(mg/100 cm^2^·d)**
24.6	4842	2154	0.0494	0.0248
**Annual Mean** **Relative Humidity (%)**	**Rainfall** **(mm/a)**	**Climate Type**	**Cl^−^** **(mg/100 cm^2^·d)**	**NO_2_** **(mg/100 cm^2^·d)**
86	1942	Marine	0.7695	0.0091

**Table 4:** Electrochemical parameters calculated from potentiodynamic polarization curves.

Corrosion Time (h)	0	48	96	144	288	432	576
***i*_corr_** (μA/cm^2^)	63.1975	52.6623	98.4011	84.3141	27.4536	49.5678	59.2107
***E*_corr_** (V)	−1.0506	−0.8942	−0.8156	−0.8480	−0.7690	−0.7136	−0.5983
***β*_a_** (mV/decade)	150	162	179	203	218	217	234
***β*_c_** (mV/decade)	−76	−133	−198	−183	−177	−210	−303
***Rp*** (Ω/cm^2^)	347.03	603.00	415.38	496.29	1547.06	936.10	969.52

**Table 5:** Fitted EIS parameters for the specimens after different corrosion periods.

**Corrosion Time** (h)	0	48	96	144	288	432	576
***R*_s_**(Ω·cm^2^)	5.703	1.184	9.887	3.346	9.394	4.481	5.214
***Q*_r_** (μF·cm^−2^)	-	-	-	117.6	69.84	24.27	20.53
***n*_r_**	-	-	-	0.4831	0.3957	0.3998	0.4037
***R*_r_**(Ω·cm^2^)	-	-	-	4.754	38.94	111.7	153.5
***Q*_dl_**(mF·cm^−2^)	13.87	5.504	22.46	30.36	6.382	3.256	2.492
***n*_dl_**	0.4855	0.1724	0.3257	0.3734	0.3084	0.3023	0.3088
***R*_ct_**(Ω·cm^2^)	313.3	67.8	27.18	125.2	176.4	265.3	427.7
***Y*_0_**(Ω·cm^−2^·s^1/2^)	-	1.22 × 10^−3^	5.411 × 10^−3^	1.462 × 10^−4^	5.228 × 10^−4^	1.92 × 10^−4^	2.633 × 10^−4^
***χ* ^2^**	2.633 × 10^−3^	4.881 × 10^−4^	7.449 × 10^−5^	2.719 × 10^−5^	4.127 × 10^−4^	1.157 × 10^−4^	7.882 × 10^−5^

## Data Availability

Not applicable.
